# Deaths from surgical conditions in Malawi - a randomised cross-sectional Nationwide household survey

**DOI:** 10.1186/s12889-020-09575-8

**Published:** 2020-09-25

**Authors:** Carlos Varela, Sven Young, Reinou S. Groen, Leonard Banza, Nyengo Mkandawire, Bente Elisabeth Moen, Asgaut Viste

**Affiliations:** 1 Department of Surgery, Kamuzu Central Hospital, Lilongwe, Malawi; 2 Department of Surgery, University of Malawi, College of Medicine, Lilongwe, Malawi; 3 Department of Clinical Medicine, Centre for International Health, Department of Global Public Health and Primary Care, University of Bergen, Bergen, Norway; 4 Department of Orthopaedic Surgery, Haukeland University Hospital, Bergen, Norway; 5 Department of Obstetrics and Gynaecology, Johns Hopkins Medicine, Baltimore, USA; 6 Department of Obstetrics and Gynaecology, Alaska Native Medical Centre, Anchorage, USA; 7 Department of Surgery, University of Malawi, College of Medicine, Blantyre, Malawi; 8 Department of Research and Development, Haukeland University Hospital, Bergen, Norway

**Keywords:** Surgical, Deaths, National survey, SOSAS

## Abstract

**Background:**

Relatively little is known about deaths from surgical conditions in low- and middle- income African countries. The prevalence of untreated surgical conditions in Malawi has previously been estimated at 35%, with 24% of the total deaths associated with untreated surgical conditions. In this study, we wished to analyse the causes of deaths related to surgical disease in Malawi and where the deaths took place; at or outside a health facility.

**Methods:**

The study is based on data collected in a randomised multi-stage cross-sectional national household survey, which was carried out using the Surgeons Overseas Assessment of Surgical Need (SOSAS) tool. Randomisation was done on 48,233 settlements, using 55 villages from each district as data collection sites. Two to four households were randomly selected from each village. Two members from each household were interviewed. A total of 1479 households (2909 interviewees) across the whole country were visited as part of the survey.

**Results:**

The survey data showed that in 2016, the total number of reported deaths from all causes was 616 in the 1479 households visited. Data related to cause of death were available for 558 persons (52.7% male). Surgical conditions accounted for 26.9% of these deaths. The conditions mostly associated with the 150 surgical deaths were body masses, injuries, and acute abdominal distension (24.3, 21.5 and 18.0% respectively). 12 women died from child delivery complications. Significantly more deaths from surgical conditions or injuries (55.3%) occurred outside a health facility compared to 43.6% of deaths from other medical conditions, (*p* = 0.0047). 82.3% of people that died sought formal health care and 12.9% visited a traditional healer additionally prior to their death. 17.7% received no health care at all. Of 150 deaths from potentially treatable surgical conditions, only 21.3% received surgical care.

**Conclusion:**

In Malawi, a large proportion of deaths from possible surgical conditions occur outside a health facility. Conditions associated with surgical death were body masses, acute abdominal distention and injuries. These findings indicate an urgent need for scale up of surgical services at all health care levels in Malawi.

## Background

Surgically treatable conditions are a major contributor to the global burden of disease. It is estimated that 11–15% of the global burden of disease, measured as disability-adjusted life years (DALYs), could be treated and corrected surgically [1, 2]. Surgical diseases account for up to 15% of the total DALYs lost globally, or 38 DALYs lost per 1000 people per year [3]. Shrime et al. estimated that of the total deaths globally, 31.3% are related to surgical conditions, and 25.1% of DALYs were lost due to surgical conditions in LMICs [4]. Untreated surgical conditions could contribute to 20% of the deaths affecting young adults in LMICs and to about 10% of all deaths globally [2, 3]. Studies from several sub-Saharan African countries have found a prevalence of 6 to 35% for conditions needing surgical consultation or treatment [5–8].

Surgery has been described as the neglected stepchild of global health. Though there are more studies being done to map the burden of disease, there are few surgical care providers in low- and middle-income countries (LMICs) and low priority is given to surgical conditions by public health care systems [2]. Surgical need assessments in LMIC have shown that injuries, malignancies, congenital anomalies, complications of pregnancy and cataracts are the most predominant conditions requiring surgery [1, 9]. In addition, over 100 million people sustain traumatic injuries globally each year. Of these, more than 5 million people die from their injuries. This is more deaths than from HIV, malaria, and tuberculosis combined. About 90% of these take place in low- and middle-income countries (LMICs) [4]. Deaths and morbidity from surgery-related diseases in LMICs can be reduced by scaling up basic, life-saving surgical care [6, 7].

Malawi is a low income country in south-eastern Africa with limited access to surgical care, especially for the majority rural population [5, 10]. We have previously estimated the proportion of deaths from untreated surgical conditions in Malawi including trauma to be around 24% of all deaths [5]. In this country wide cluster-randomised household survey, more than a third of the population was living with a condition that needed a surgical consultation or treatment. However, little is known about what injuries and surgical conditions result in death in Malawi, and what proportion is likely to be due to lack of access to surgical care.

Reducing deaths from trauma and other surgical conditions, requires knowledge of where the deaths occur, cause of death and what barriers there are to accessing surgical care [11, 12]. The aim of this study, therefore, was to outline the causes and location of deaths from untreated surgical conditions and trauma in Malawi.

## Methods

### Setting

This study is part of a larger project assessing the unmet surgical needs in Malawi [5, 13]. The research setting has previously been described in detail in the two related previous publications on untreated surgical conditions and transportation barriers in Malawi [5, 13]. The population of Malawi is 18.4 million [10], with a GNI per capita of USD 340 (World Bank Group, 2016) [14]. The country is divided into three geographic regions and 28 administrative districts. There are over 48,000 registered settlements [10], the majority of which are located in the rural areas with poor road infrastructure and limited access to health care [13] .

### Survey instrument

Surgeons OverSeas (SOS) have developed the Surgeons OverSeas Assessment of Surgical need (SOSAS) enhanced verbal autopsy tool [6, 8, 15, 16]. SOS granted permission to use and adapt the SOSAS survey tool to suit the Malawi setting. It was translated into the official local language, Chichewa, and was installed on portable electronic tablets that were used by trained data collectors [5, 13].

The SOSAS tool consisted of a questionnaire with three components; one for general household information, and forms for two individual household members who were identified by randomisation of the members of the household. The verbal autopsy included an inquiry on the number of people who died in the household in the 12 months prior to the interview, their gender and age and cause of death. Identification of a household member with the following conditions in the week before they died were considered to be surgical: bleeding or illness during child birth; abdominal distention or pain associated with vomiting and not passing stool; mass, swelling or growth e.g. breast tumours and limb tumours; Injury; acquired deformity; or wound not due to injury, or, in neonates; congenital deformity including conditions of any visible abnormality, feeding problems, problems with urinating or passing stool soon after birth.

Further, data were collected about duration of illness, place of death (home, health facility and elsewhere), and initial care. (SOSAS tool page 5 and 6, *www.surgeonoverseas.org/resources/*,. Causes of death not specified were recorded as other medical conditions.

### Pilot study and sample

A pilot study was done in rural areas of the capital city, Lilongwe, in February 2016, to validate the data collection tool in a Malawian set up. Fifteen third year medical students from the University of Malawi, College of Medicine were used as data collectors to interview 100 households, and 200 people in four different settlements within Lilongwe. The pilot study confirmed that the electronic questionnaire functioned well and gave an estimated prevalence of surgical conditions of 25%. This figure was used to estimate sample size for the national survey (5).

The needed study population was estimated to be 1497 households national wide with 2994 (95% CI) interviewees and a design effect of 1.5. The Malawian National Statistics Office provided a list of all enumeration areas from the Malawi Census Board based on the 2008 national census records [10]. From the 28 administrative districts, 55 settlements were randomly selected using computer generated random numbers. From each settlement, 2–4 households were selected randomly depending on the size of the settlement i.e. 2 in smaller settlements and 4 in larger settlements. From each household, the head of the household and another randomly selected person were interviewed.

### Data collection

**Fig. 1:**
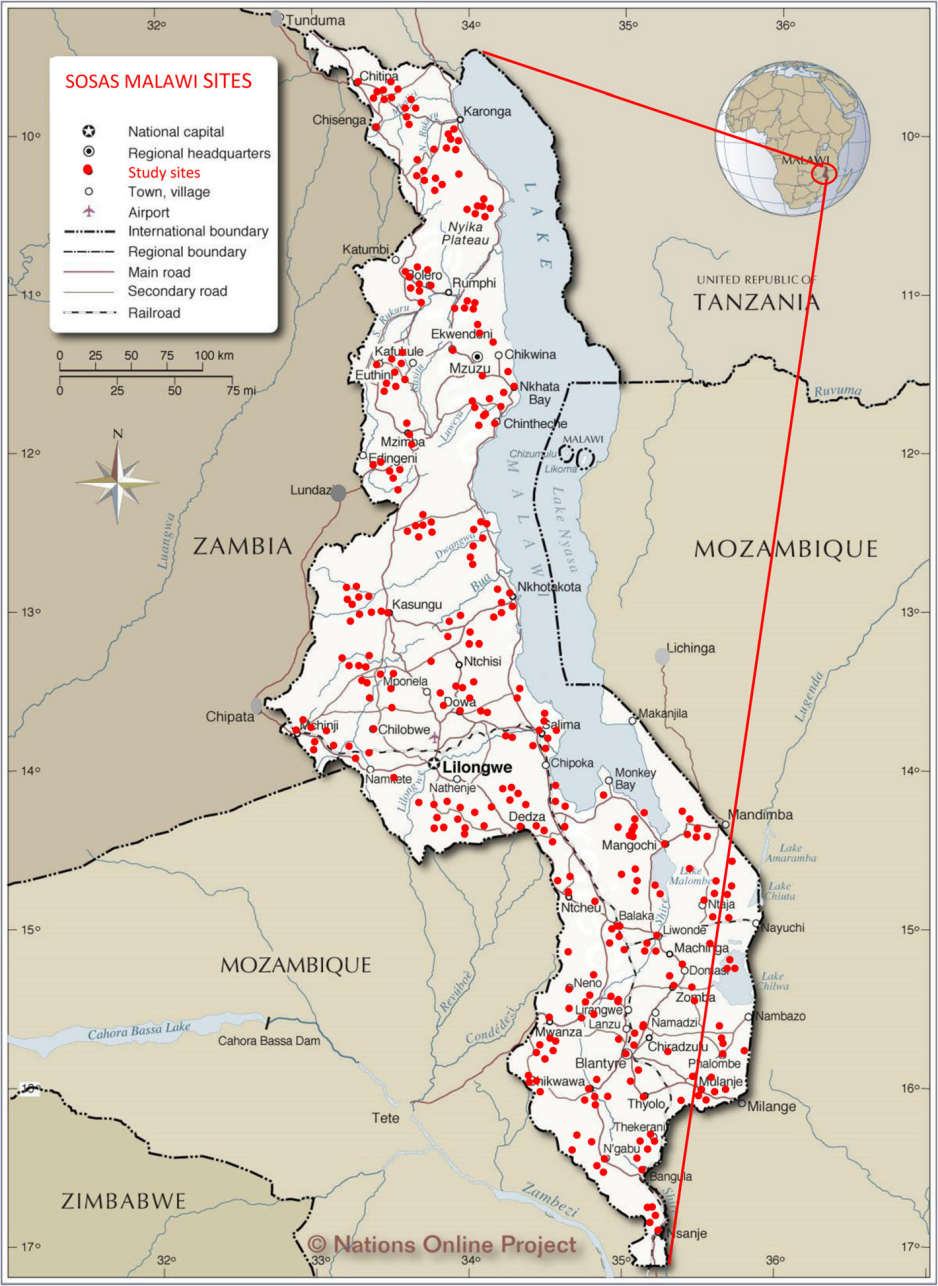
Map of study sites. Study sites for SOSAS Malawi survey on unmet surgical need. Reprinted with permission from Malawi Medical Journal; MMJ 29(3)231–236 Sept 2017)

Thirty-two 3rd year medical students received a 10 days training course as data collectors before the pilot study, and a 5 days refresher course was conducted prior to the national survey. It took this team of data collectors (led by authors CV and LB) 10 weeks in July and August 2016 to visit the settlements randomised for inclusion (Fig. 1*; reprinted with permission from Malawi Medical Journal; MMJ 29(3)231–236 Sept 2017*). 27 of 28 districts were visited. It was not possible to reach the remote Likoma Island district in Lake Malawi, due to time and funding constraints.

Due to language barriers in some rural areas with different local languages, some interviews were performed by use of translators. Data was exported directly into an Excel (Microsoft 2010) data-base on a computer at the end of each day for data security and to spot check data quality.

### Data analysis

Data analysis was done using SPSS version 24. Pearson’s Chi-square test was used to test the difference in rates in two different groups.

## Results

A total of 1479 households were included in the study. 1332 (90.1%) of the households were in rural areas. The median household size was 6 persons, and median age was 22 years. The total number of deaths reported due to all causes comprising surgical and medical conditions was 616. 58 entries did not have sufficient data on cause of death. 558 deaths were available for further evaluation. Of these, 150 (26.9%) were assessed as being caused by a surgical condition or injury. 12 deaths (8.3% of surgical deaths) were women in the age group 18–49 who died due to bleeding related to childbirth (Table 2). Children below 18 years represented 45.7% (255 persons) of the recorded deaths and 33.8% were below the age of 5 years (Table 1).

**Table 1:** Household deaths in preceding 12 months

	Frequency (n)	(%)
**House hold location**		
Rural	1332	90.1
Urban	107	7.2
Slum	13	0.9
Not recorded	27	1.8
**Total households**	**1479**	***100***
Total Reported deaths		
Male	294	52.7
Females	259	46.4
Not recorded	5	0.8
All	558	100
Total Deaths by age group (years)		
0–4	188	33.8
5–17	67	12.1
18–49	172	31.0
Above 50	128	23.1
Missing	3	
Total	558	100

Health care was sought by 459 persons (82.3%) before their death, whereas 99 persons (17.7%) did not contact a health facility for medical care. There was 12.9% that additionally visited a traditional herbalist prior to a health facility. Of the people that died from assumed surgical conditions 32 (21.3%) received surgical care (11.3% major, 10% minor surgical intervention.) Household heads reported that, out of the 459 persons who sought health care, 284 (62.1%) of the total deaths went to a local health facility, while 59 (12.9% of all death) went to a traditional herbal medicine provider additionally for consultation and traditional medicine treatment, and the rest visited other distant health care facilities.

Of the 150 cases defined as related to surgical conditions (24.0% of total deaths), 35 (24.3%) were described as having a swelling or mass, 31 (21.5%) were related to injury and 27 (18.0%) were due to a condition with acute abdominal distension of less than 1 week duration (Table 2). Of the 31 injuries, 16 (51.6%) were related to road traffic accidents. The majority of these deaths (14 persons – 45.2%) occurred in the age group 18–49 (Table 3).

**Table 2:** Causes of death in the past year according to age

	Injury	Infected wound	Bleed from child birth	Regional Body mass	Congenital deformity	Acquired deformity	Acute Abdominal distention	Surgical deaths	Non-surgical/ Medical
**0–4 yrs**	1	3	0	7	11	0	5	**27**	152
**5–17 yrs**	3	1	1	4	2	0	7	**18**	49
**18–49 yrs**	14	8	10	10	0	5	13	**60**	115
**Above 50 yrs**	13	3	1	14	2	4	2	**39**	88
**Unknown age**	0	2	0	0	1	3	0	**6**	4
**Surgical deaths:** **n (%)**	**31 (21.5%)**	**15 (10.4%)**	**12 (8.3%)**	**35 (24.3%)**	**15 (10.4%)**	**9 (6.3%)**	**27 (18.0%)**	**150 (26.9%)**	**408 (73.1%**

Total deaths 150, 10 missing data for age

**Table 3:** Deaths due to trauma

	Bite/animal attack	Burn/explosion	Traffic related injuries	Fall from height	Stab/slash	Death age group n (%)
**0–4 yrs**	0	1	0	0	0	**1 (3.2%)**
**5–17 yrs**	0	1	1	1	1	**4 (12.9%)**
**18–49 yrs**	1	0	8	1	4	**14 (45.2%)**
**Above 50 yrs**	1	0	7	2	2	**12 (38.7%)**
**Deaths from traumatic cause n (%)** **(*N*) = 31**	2 **(6.5%)**	2 **(6.5%)**	16 **(51.6%)**	4 **(12.9%)**	7 **(22.5%)**	31 **(100%)**

^a^Responses from household members in reply to the question: … .“condolences for your loss … ..which one of the above injuries may have caused the death of your family member?

55.3% of probable surgical deaths occurred outside a healthy facility, compared to 43.6% of other deaths *(p = 0.00473).* (Table 4)

**Table 4:** Location of death for persons that died during the last year

	At health Facility	At home	Other location	Unknown location	Total
**Deaths related to probable surgical condition**	61 (10.9%)	67 (12.0%)	16 (2.9%)	6 (1.1)	150 (26.9%)
**Other deaths/medical**	227 (40.7%)	164 (29.4%)	14 (2.5%)	4 (0.7)	408 (73.1%)
**Total deaths**	288 (51.6%)	231 (41.4%)	30 (5.4%)	10 (1.8)	558 (100%)

Total deaths 558, 10 missing data for location

^a^Response of household head” … . Condolences for your loss, where did your family member die from one of the above locations …?”

## Discussion

This study has shown that 55.3% of deaths due to surgical conditions in Malawi occurred outside a health facility. This is higher than for deaths due to other medical conditions. Most of the surgical deaths outside health facilities happened at home. There are few studies on this topic, as most studies of surgical patients are based on hospitalized patients. However, a large study of 80,483 women of reproductive age in Mozambique indicated that 61.1% of deaths of women occurred at a health facility, 27.8% at home and 11.1% occurred somewhere else (for example on way to a health facility). These figures indicate similar problems as seen in the present study [17]. The difference in proportion of deaths happening outside a health facility, between surgical related and non-surgical related deaths can be partially explained by the fact that some road traffic injuries will lead to death at the site of injury. However, this finding still highlights a large lack of capacity for transport and health care in Malawi.

The problem with lack of access to surgery and trauma centres in low income settings has also been described in a study on acute abdominal conditions and other emergency conditions in India and lack of emergency obstetric services in Mozambique [18, 19]. A study done in Zambia demonstrated that only 16.5% of the hospitals met the WHO minimum standards of safe surgical care [20]. There is a similar situation in Malawi with low standards of safe surgery in rural health facilities [21]. In addition, the need of improvement in global surgical care, particularly in low- income and middle- income countries is described in a review from 2019 [21].

Some communities are remote, with large distances to health facilities, and family members may not have enough financial resources to help with transportation to the hospital [13]. This presents a further delay, or barrier, to patients being able to present to the health facility, which in many cases leads to loss of life. People in the rural communities of Malawi often visit a traditional healer before considering visiting a formal health care facility [22]. In our study, 12.9% of patients that died went to a traditional healer prior to visiting a formal health facility, possibly contributing to delayed presentation for surgical health care. Not all surgical conditions need operative interventions, but access to consultation with qualified health personnel can help identify those at risk and the need of surgical intervention or non-operative management.

The present study shows that surgical conditions that significantly affect mortality in Malawi were found to be 26.9% of all deaths. Overall the dominant causes of death were localised body masses, like breast mass, extremity masses and other body torso masses, representing such conditions as tumours, abscesses and hernias. Acute abdominal distension and traumatic conditions were the other dominant causes of untreated surgical condition related deaths. Though abdominal distension can arise from other medical conditions, in this survey death related to abdominal distension was defined by the interviewer as an acute death occurring within 1 week of the abdominal distension. This condition is highly suspicious of a surgical condition e.g. bowel obstruction or bowel perforation with peritonitis. There is little literature on this from Malawi. A previous study at a referral hospital in Lilongwe showed that the common aetiology for peritonitis were appendicitis and intestinal volvulus. It was also found that 11% of acute abdominal presentation with peritonitis was due to perforated peptic ulcer and small intestinal perforation respectively, and mortality from this was 15% [23]. The complications of untreated surgical conditions like bowel perforation, gangrene, dehydration and respiratory compromise can result in high morbidity and mortality rates. A study in East Africa reported morbidity rate of 24% and a mortality rate of 12.9% from abdominal surgical conditions due to bowel obstruction [24].

Children, below the age of 5, represented 18.8% of the surgical deaths with the majority of them dying from congenital disorders (40.7%). A study in a paediatric population in Malawi from 2016, reported a mortality rate of 23.3% in neonates due to different kinds of intestinal obstruction, most of which were congenital [25]. This study also showed that, in children, congenital surgical conditions, such as Hirschsprung’s disease and anorectal malformations, accounted for 29 and 18.5% of intestinal obstructions in neonate respectively [25]. This is associated with high mortality if not diagnosed promptly and treated properly in time by surgical intervention. Similarly, a Kenyan study showed that the highest mortality rates among neonates and infants were related to acute abdomen, 7% of congenital deaths [24]. In our study there were 11 neonates that died from congenital surgical conditions after being born alive. This survey did not investigate the burden of still birth, as these are culturally not registered as part of the population in Malawi. Congenital intestinal obstruction in neonates constitutes a major portion of neonatal surgical problems. Similar to our study, the Kenyan study was based on information from households. However, the Kenyan study used other categories for the reasons for death and had a longer observation period [24].

Trauma is another leading cause of death, and in our study it contributed to 21.5% of all surgical deaths, mainly in the age group 18–49. There were 16 persons (51.6% of traumatic deaths) that died from traffic related injuries. This reflects findings from another study in Malawi that showed a rapid rise in road traffic injuries in Malawi from 2009 to 2015 [26].

We registered 12 women who died during pregnancy and childbirth. However, the specific cause of death was in most cases not documented, except that bleeding was reported. Death from child birth complications was due to excessive haemorrhage associated with child delivery, i.e. post-partum haemorrhage. The Maternal Mortality Ratio in Malawi has been estimated at 675 maternal deaths/100000 live births during the period 2004–2010 [20]. It should be noted that this figure is far from Sustainable Development Goal 3.1, where the aim is to reduce maternal mortality to less than 70 per 100,000 live births (www.who.int/sdg/targets/en/).

A study done in Malawi assessing maternal mortality from delays in accessing obstetric medical care showed that the cost of transport and insufficient family finances, poor road conditions or terrain, shortage of health workers and providers, long travel to the nearest health facility and an inadequate referral system contributed significantly to delays in timely obstetric care. In this study 62.2% of maternal deaths occurred at a health facility while 21.2% of the deaths happened at home [27]. Improving health facility systems and implementing models like “saving mothers, giving life” (SMGL) initiatives may help to reduce deaths that happen from acute obstetric complications at rural or primary health care centres [28].

The absence of appropriate surgical care in LMICs results in many unnecessary deaths from curable surgical conditions. This lack of services contributes to significant disability, economic loss and ultimately compromises the quality of life for people living in these regions. Key barriers to accessing surgical services are; cost of transport, distance, poor roads, and lack of suitable transport [13]. Most people present late to health facilities as a result of the different transportation barriers they have faced [13]. Cultural issues like consulting the traditional herbalist for traditional medical intervention might also delay timely surgical intervention.

A limitation of this study was that the information of the causes of death was limited, since in many cases, no clear diagnosis was given. Data relies on the medical understanding of the informant, and this is likely to have weaknesses. However, in a validation study in Nepal, the SOSAS survey was compared with a visual examination and demonstrated high concordance with the self-reports from the participants [29]. Another limitation is that the information might be hampered by recall bias, with the informant thinking back in time over the past year, as well as specific causes of the events surrounding the deaths. However, this study also has many strengths, most obviously its sample size, response rate and covering nearly the whole geographical area of Malawi through randomization of survey sites. The interviewers were skilled and specifically trained for the study, and they used a standardized interview guide developed specifically to assess surgical need. Interviews were chosen because other sources for this information were not available in Malawi. Also, questionnaires were not an option, due to a moderately high illiteracy rate in Malawi [30], and a lack of culture for this type of data gathering.

## Conclusion

In Malawi, deaths due to probable surgical causes were characterised by body swellings or tumours, acute abdominal distention and injuries. Over half the deaths from surgical conditions occurred outside a health facility, significantly more than seen for non-surgical conditions. This indicates an urgent need for scale up of surgical services at all health care levels in Malawi.

## Data Availability

The data sets used and analyses during the study are available from the corresponding author on reasonable request.

## Abbreviations

CAM: Complementary and Alternative Medicine
CI: Confidence Interval
DALYs: Disability Adjusted Life Years
EHP: Essential Health Package
LIC: Low income countries
LMIC: Low and middle income countries
MMR: Maternal Mortality Ratio
NGO: Non-Government Organisation
NORAD: Norwegian Programme for Capacity Development in Higher Education and Research for Development
SOSAS: Surgeons Overseas Assessment of Surgical need
SPSS: Statistical Package for Social Scientists
SSA: Sub – Saharan Africa
WHO: World Health Organisation
